# MBFILNet: A Multi-Branch Detection Network for Autonomous Mining Trucks in Dusty Environments

**DOI:** 10.3390/s25175324

**Published:** 2025-08-27

**Authors:** Fei-Xiang Xu, Di-Long Zhu, Yu-Peng Hu, Rui Zhang, Chen Zhou

**Affiliations:** 1 School of Information and Control Engineering, China University of Mining and Technology, Xuzhou 221116, China; xufx92@cumt.edu.cn (F.-X.X.); zdl0130@cumt.edu.cn (D.-L.Z.); hyphyp_18@cumt.edu.cn (Y.-P.H.); zrcumt@cumt.edu.cn (R.Z.); 2 State Key Laboratory of Fluid Power & Mechatronic Systems, Zhejiang University, Hangzhou 310027, China; 3 Guangdong Institute of Electronic Information Engineering, University of Electronic Science and Technology of China, Dongguan 523950, China

**Keywords:** autonomous mining truck, object detection, dusty weather, multi-branch feature interaction

## Abstract

As a critical technology of autonomous mining trucks, object detection directly determines system safety and operational reliability. However, autonomous mining trucks often work in dusty open-pit environments, in which dusty interference significantly degrades the accuracy of object detection. To overcome the problem mentioned above, a multi-branch feature interaction and location detection network (MBFILNet) is proposed in this study, consisting of multi-branch feature interaction with differential operation (MBFI-DO) and depthwise separable convolution-enhanced non-local attention (DSC-NLA). On one hand, MBFI-DO not only strengthens the extraction of channel-wise semantic features but also improves the representation of salient features of images with dusty interference. On the other hand, DSC-NLA is used to capture long-range spatial dependencies to focus on target-object structural information. Furthermore, a custom dataset called Dusty Open-pit Mining (DOM) is constructed, which is augmented using a cycle-consistent generative adversarial network (CycleGAN). Finally, a large number of experiments based on DOM are conducted to evaluate the performance of MBFILNet in dusty open-pit environments. The results show that MBFILNet achieves a mean Average Precision (mAP) of 72.0% based on the DOM dataset, representing a 1.3% increase compared to the Featenhancer model. Moreover, in comparison with YOLOv8, there is an astounding 2% increase in the mAP based on MBFILNet, demonstrating detection accuracy in dusty open-pit environments can be effectively improved with the method proposed in this paper.

## 1. Introduction

With the rapid development of intelligent mine construction, autonomous mining trucks (AMTs) have been widely applied in mining, owing to their advantages in fatigue driving risk mitigation and operational cost reduction [1]. It is important for AMTs to perceive surrounding environmental information, which is the foundation of decision-making and control. As a common evaluation indicator of perception, high object detection accuracy plays a vital role for AMTs in complex mining environments [2]. Although object detection for urban road scenarios has reached maturity, its application in unstructured mining environments remains remarkably limited.

Object detection is tough in open-pit mines due to the complexity of associated environments due to, e.g., significant scale variation, severe mutual occlusion, and the camouflage effect. To address the problem of significant scale variation, Song et al. [3] built MSFANet to capture rich context features, enhancing the feature saliency of objects with different scales. Simultaneously, detection accuracy is also decreased, owing to the loss of critical feature information, which is caused by mutual occlusion between objects in open-pit mines. Therefore, Bo et al. [4] combined the guidance module of contextual information with the efficient squeeze–excitation attention mechanism, ensuring the model focuses on channels with important feature information. In addition, due to interference caused by the high similarity between the target and background, missed and misused results are increased during detection. Hence, Ren et al. [5] constructed a multi-scales fusion and attention-based model to improve the performance of object detection for camouflaged obstacles in mining.

**Figure 1:**
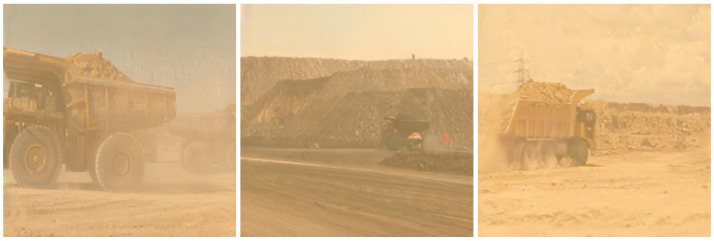
Dusty images in open-pit mine.

Apart from the aforementioned characteristics in unstructured open-pit mine environments, variable weather is also a important factor affecting the accuracy of object detection. Currently, research on object detection for AMTs primarily focuses on normal weather conditions, but AMTs often operate in dusty environments [6], as shown in Figure 1. Compared with normal weather conditions, visual characteristics of images such as color balance, fine-grained details, and luminance are severely distorted by dust interference, complicating the extraction of critical channel-wise semantic features and the representation of salient object information [7,8,9]. Ordinary bad weather (such as rain, fog, etc.) is evenly distributed in the form of droplets and introduces interference based on Mie scattering. In contrast with ordinary bad weather, mineral dust is composed of irregularly shaped mineral particles with an uneven spatial distribution, causing local occlusion that disrupts object feature continuity and induces edge confusion in detection tasks. Additionally, current detection of adverse weather conditions is based on the principle of uniform distribution of rain, fog, and water droplets in the atmosphere, aiming to enhance detection performance by reducing optical interference. However, the distribution of dust particles in dusty environments violates this principle, and at the same time, dust particles can block the texture and edge details of objects, making existing detection algorithms inapplicable. Subsequently, inaccurate positioningand false detection may occur due to such complications, directly impairing the detection accuracy of AMTs [10].

In this article, an efficient detector networkfor AMTs is presented to specifically addresses the robust extraction of salient target features and accurate identification of structural characteristics, thereby improving detection accuracy for unmanned mining trucks in dusty environments. The main contributions of this work are as described as follows:

(1) To improve the accuracy of object detection for AMTs in dusty weather, a novel object detection method based on YOLOv8 and named multi-branch feature interaction and location detection net (MBFILNet) is presented.

(2) Aiming at the challenge of discriminating salient target information in dust-laden environments, the dust interference is filtered out based on MBFI-DO, which integrates multi-branch information interaction and employs differential information guidance.

(3) To enhance the discrimination of structural features in dust-obscured environments, DSC-NLA is designed to capture global spatial long-range dependencies and enhance cross-channel information interaction. This design can augment the recognition robustness of detection models across different dust levels.

The remainder of this research is organized as follows. Related works on detection under adverse weather conditions are introduced in Section 2. The proposed MBFILNet is elaborated upon in Section 3. In Section 4, a large number of experiments on DOM are conducted to evaluate the performance of MBFILNet. Finally, conclusions are presented in Section 5.

## 2. Related Works

In this section, relevant works about object detection under adverse weather, as well as feature enhancement and global context feature representation, are analyzed briefly.

### 2.1. Object Detection Under Adverse Weather

Although the YOLO series [11] and Faster R-CNN [12] methods achieve high detection accuracy in optimal weather conditions, their performance proves less satisfactory under adverse weather phenomena [13]. To overcome this problem, research in the last few years has primarily focused on three approaches: (1) image restoration networks, (2) enhanced detection algorithms, and (3) domain adaptation. Restoring an image is conducive to improving the image quality, enhancing the detection accuracy. Huang et al. [14] developed DSNet, a dual-branch network sharing feature extraction for joint image restoration and detection. IA-YOLO [15] comprises restoration and detection networks with a learnable image processing module, which is trained in an end-to-end manner using detection loss alone. However, the approaches mentioned above inevitably lose critical image information during the recovery process. To mitigate information loss in restoration networks, enhanced detection algorithms with scene feature extraction capabilities have been proposed. A lightweight object detection network with a novel plug-and-play Cross-Fusion (CF) block was proposed by Ding et al. [16], combining the advantages of FPN and PAN in a more flexible architecture. Conventional detection algorithms require a substantial amount of training data, yet datasets under adverse weather conditions are particularly limited. To address these limitations, domain adaptation techniques have attracted widespread attention in the field of image detection. Wang et al. [17] proposed the image Quality Translation Network (QTNet) and Feature Calibration Network (FCNet), enabling models to progressively generalize from clear-weather to adverse-weather domains based on domain adaptation. Nevertheless, cross-domain learning often loses critical channel dimension information, resulting in poor accuracy performance under adverse weather conditions.

Furthermore, dust storms occur less frequently compared to traditional adverse weather conditions, and they are mostly found in open-pit mines, resulting in a scarcity of related research. Currently, there are two kinds of approaches for object detection in dusty mines: image restoration methods and enhanced detection algorithms. For example, TIAN et al. [18] proposed a coal mine image enhancement algorithm based on dual-domain decomposition, which achieved image defogging in low-frequency images to eliminate the influence of dust. However, this method ignores the color-shift effect caused by coal dust, resulting in color distortion of the restored image and an unsatisfactory dust removal effect. In terms of enhanced detection algorithms, Yang et al. [19] proposed a multi-scale edge enhancement (MSEE) module and fused it with the C2f module, which enhanced the extraction of the personnel feature under high-dust conditions. Nevertheless, this approach failed to address the background interference of dust without significant improvement of detection accuracy. In summary, there is a significant gap in the research on dusty open-pit mines, so we need to conduct research on the detection of mines in dusty environments.

Motivated by enhanced detection algorithms, in this paper, crucial latent features are preserved by analyzing images corrupted by dust, thereby avoiding the information loss inherent in preprocessing stages.

### 2.2. Feature Enhancement

Deep learning-based object detection methods extract high-dimensional features through backbone networks, but the feature extraction abilities are reduced in dust-obscured images due to interference from backgrounds. To enhance multi-scale representation, Lin et al. [20] introduced the Feature Pyramid Network (FPN) by aggregating high-level and low-level features across different resolutions. However, this approach failed to address feature degradation in adverse weather conditions, limiting its robustness in adverse environments. To enhance feature representation in weather-degraded scenarios, Chen et al. [21] proposed detail-enhanced convolution (DEConv) and content-guided attention (CGA), boosting dehazing performance significantly. But the methods mentioned above are sufficient for specific tasks only an cannot achieve detection in dynamic and changing environments. To adapt to changing environments, Li et al. [22] developed a change detection difference enhancement module to extract critical features from difference maps. Based on the above analysis, it can be seen that existing methods primarily focus on detailed feature extraction, neglecting salient feature enhancement through background interference suppression.

In order to address this limitation, in this paper, we propose MBFI-DO, which guides discriminative feature extraction in target regions based on the the integration of high-level semantic information, suppressing dust interference while enhancing salient feature representation.

### 2.3. Global Context Feature Representation

Enhancing the feature representation in dusty environments [23,24] is crucial for target localization based on global receptive fields and contextual information, which can be obtained by modeling the global relationship between targets and the background [25,26]. Noman M et al. [27] constructed an efficient local–global context aggregator (ELGCA) module within the encoder to capture enhanced global context and local spatial information. However, oversimplification of long-range dependencies may occur due to its fixed pooling strategy. To address the challenge of feature refinement, Hu et al. [28] designed a guided refinement decoder (GRD) to extract context information and further refine prediction results. Nevertheless, dust-induced noise is propagated from high-level features to finer scales due to the lack of adaptive filtering. In contrast, the non-local neural network (NLNet) proposed in ref. [29] established reliable global context feature representations by calculating the pairwise correlations between spatial pixels, in which noise interference is reduced at the same time.

Inspired by the theoretical insights in ref. [29], DSC-NLA is proposed in this paper. First, the multi-scale receptive field feature from feature maps is extracted by means of depthwise separable convolution. Subsequently, the representations of the heterogeneous receptive field are aggregated based on feature fusion strategies. Finally, pixel-wise correlation modeling is employed to construct spatial long-range dependencies, and target localization is finished.

## 3. Methods

In this section, the overall architecture of MBFILNet is introduced. Section 3.2 introduces the salient feature representation branch in detail, while Section 3.3 discusses the attention mechanism for target structural features.

### 3.1. The Overall Architecture of MBFILNet

**Figure 2:**
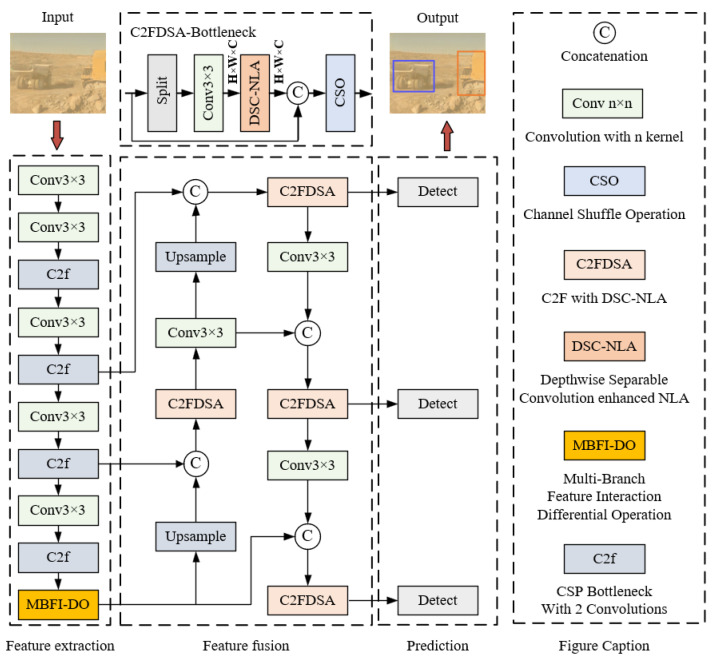
Model architecture diagram of MBFILNet.

To address the challenges of obscured salient features and lost positional information caused by background interference, MBFILNet is proposed in this paper, as illustrated in Figure 2. The proposed MBFILNet maintains the architectural framework of YOLOv8, with novel enhancements through the MBFI-DO and DSC-NLA modules. MBFI-DO enhances target feature extraction across different feature scales by implementing multi-branch cross-fusion. Then, differential operation is introduced into the model, which dynamically focuses on the extraction of discriminative target features and the mining of channel semantic information. Meanwhile, CFDSA extends the original feature fusion module in YOLOv8 C2f by adding DSC-NLA and a channel shuffle operation (CSO) [30,31]. Notably, DSC-NLA captures spatial contextual information to establish semantic correlations across regions, thereby enhancing the model’s perception of structural location features. A residual structure is also introduced to enrich the diversity of feature information in MBFILNet, which helps to alleviate the vanishing gradient problem and enables the model to learn more discriminative features.

### 3.2. Multi-Branch Feature Interaction with Differential Operation (MBFI-DO)

Substantial dust is generated during coal mining, not only hindering target recognition by even leading to missed detections [32]. To overcome this problem, a multi-branch feature interaction (MBFI) module is designed in an AMT detection system, which expands the receptive field of the backbone network and multi-branch cross-fusion. MBFI enables the model to capture multi-scale features and enhance channel-wise interaction across different feature scales. Moreover, based on the integration of differential operations (DOs), the attention of the model is dynamically guided to the extraction of discriminative target features and the mining of channel semantic information. As a result, the interference from image distortion and suspended particulate in dust-obscured environments is decoupled effectively.

**Figure 3:**
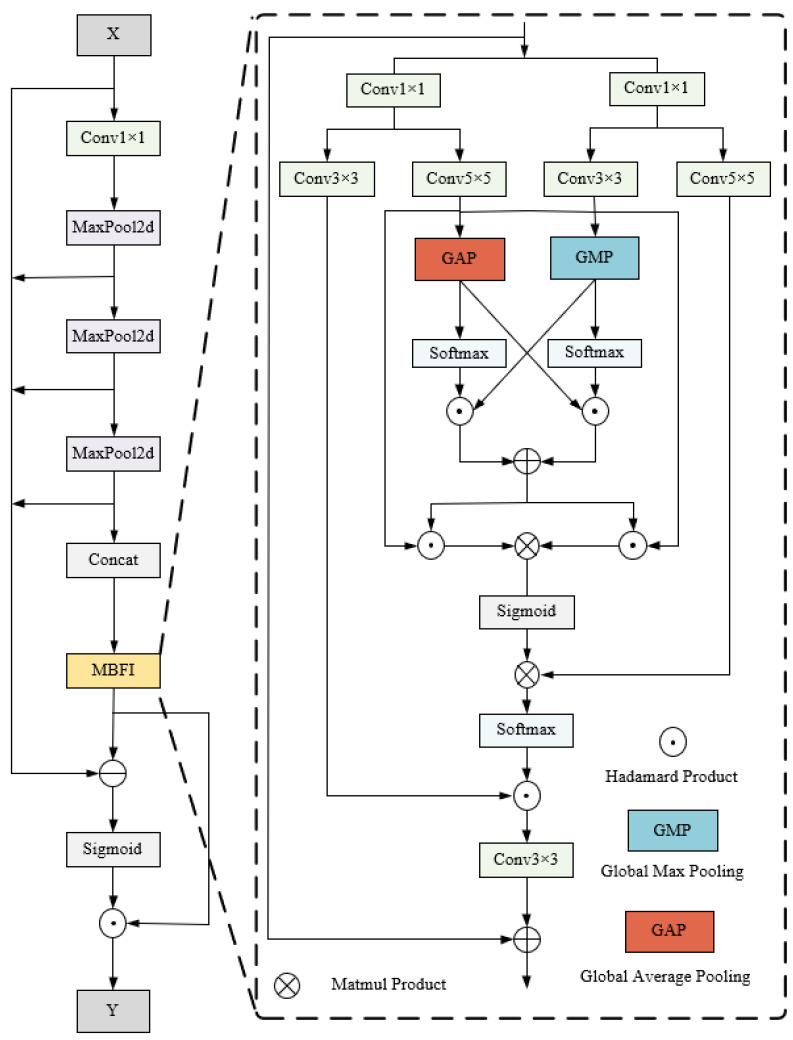
Schematic representation of MBFI-DO.

MBFI-DO not only incorporates multi-scale target feature extraction capabilities but also emphasizes channel-wise semantic information interaction, making it particularly suitable for target detection in complex, dusty open-pit mines, as illustrated in Figure 3.

A multi-path parallel architecture is designed in the MBFI module to extract rich feature information through multi-scale convolutions. Moreover, both global average pooling (GAP) and global maximum pooling (GMP) are integrated to compress features and capture channel-wise global context [32]. The mathematical expressions of GAP and GMP can be written as follows:

(1) $$Z_{c}^{a}\left(i,j\right)=\frac{1}{K\times K}\sum_{m=0}^{k-1}\sum_{n=0}^{k-1}X_{c}\left(i\times s+m,j\times s+n\right)$$

(2) $$Z_{c}^{m}\left(i,j\right)=\max_{m=0}^{k-1}\max_{n=0}^{k-1}X_{c}\left(i\times s+m,j\times s+n\right)$$

where $X_{c}$ is the input feature map and $Z_{c}^{a}$ and $Z_{c}^{m}$ represent the feature map after maximum pooling and average pooling, respectively.

Subsequently, the multi-branch fusion mechanism enhances cross-channel interaction between feature maps from diverse receptive fields, exploring inter-channel semantic correlations deeply, which can be expressed by the following formulas:

(3) $$Z^{a}=\mathrm{Softmax}\left(Z_{c}^{a}\right)\;\cdot \;f_{\mathrm{conv}\;}^{7\times 7}\left(f_{\mathrm{conv}\;}^{1\times 1}\left(X_{c}\right)\right)$$

(4) $$Z^{m}=\mathrm{Softmax}\left(Z_{c}^{m}\right)\;\cdot \;f_{\mathrm{conv}\;}^{5\times 5}\left(f_{\mathrm{conv}\;}^{1\times 1}\left(X_{c}\right)\right)$$

where Softmax denotes the normalized exponential function and $f_{\mathrm{conv}}^{1\times 1}$, $f_{\mathrm{conv}}^{3\times 3}$, $f_{\mathrm{conv}}^{5\times 5}$, and $f_{\mathrm{conv}}^{7\times 7}$ represent standard convolution operations with kernel sizes of 1 × 1, 3 × 3, 5 × 5, and 7 × 7, respectively.

Furthermore, the feature maps of rich semantic information are multiplied by the feature maps extracted from diverse receptive fields, which enhances the local salient features effectively under semantic guidance. Matrix multiplication is applied to global feature fusion, thereby jointly enhancing both spatial and channel-wise correlations across feature maps. Subsequently, a sigmoid activation function is utilized to generate the refined feature vector, as shown in Formula (5):

(5) $$Z_{0}=\sigma \left(\left(f_{con\nu }^{5\times 5}\left(f_{con\nu }^{1\times 1}\left(X_{c}\right)\right)\;\cdot \;\left(Z^{a}+Z^{m}\right)\right)\times \left(f_{con\nu }^{7\times 7}\left(f_{con\nu }^{1\times 1}\left(X_{c}\right)\right)\;\cdot \;\left(Z^{a}+Z^{m}\right)\right)\right)$$

where $Z^{a}$ and $Z^{m}$ are the resultant features of cross-multiplication. σ denotes the sigmoid activation function.

Finally, the output formula for MBFI can be written as follows:

(6) $$Y=X_{c}+\mathrm{Softmax}\left(f_{con\nu }^{1\times 1}\left(f_{con\nu }^{1\times 1}\left(X_{c}\right)\right)\;\cdot \;Z_{0}\right)\;\cdot \;f_{con\nu }^{3\times 3}\left(f_{con\nu }^{1\times 1}\left(X_{c}\right)\right)$$

The MBFI module demonstrates insufficiency for AMT detection, where simultaneous attention to both spatial information and channel-wise semantics is required. In particular, some minor but useful features are filtered out when features from different dimensions are weighted by learned attention weights.

To address the problem mentioned above, MBFI-DO is designed as a feature reuse strategy in cross-fusion feature extraction, in which MBFI is located after the concatenated max-pooling layers of SPPF; then, the feature is enhanced by differential operations (DOs). The proposed DO serves as a feature refinement mechanism that explicitly extracts and amplifies the learned feature enhancements. As a result, the representation of semantic information is strengthened through semantics-guided feature enhancement based on the MBFI-DO strategy. In detail, the feature difference between the original input feature map (*X*) and the output feature map ($F_{N}$) is calculated by the residual branch of SPPF, which is shown in Formula (7). By computing $F_{N}\left(X\right)-X$, we obtain the residual features that represent the actual improvements brought about by the MBFI module, including both channel-wise semantic reinforcements and spatial saliency information. Subsequently, multi-scale spatial–channel semantic features are extracted by multiplying the normalized difference result and $F_{N}\left(X\right)$, while dust interference is suppressed due to the enhancement of target discriminablity guided by semantic features.

(7) $$F\left(X\right)=F_{N}\left(X\right)\;\cdot \;\sigma \left(F_{N}\left(X\right)-X\right)$$

where *F* denotes the SPPF incorporated in MBFI; *X* represents the input feature map; and $F_{N}$ is composed of the four parts, namely Conv, the maximum pooling layer, concatenation layer, and MBFI.

### 3.3. Depthwise Separable Convolution-Enhanced Non-Local Attention (DSC-NLA)

**Figure 4:**
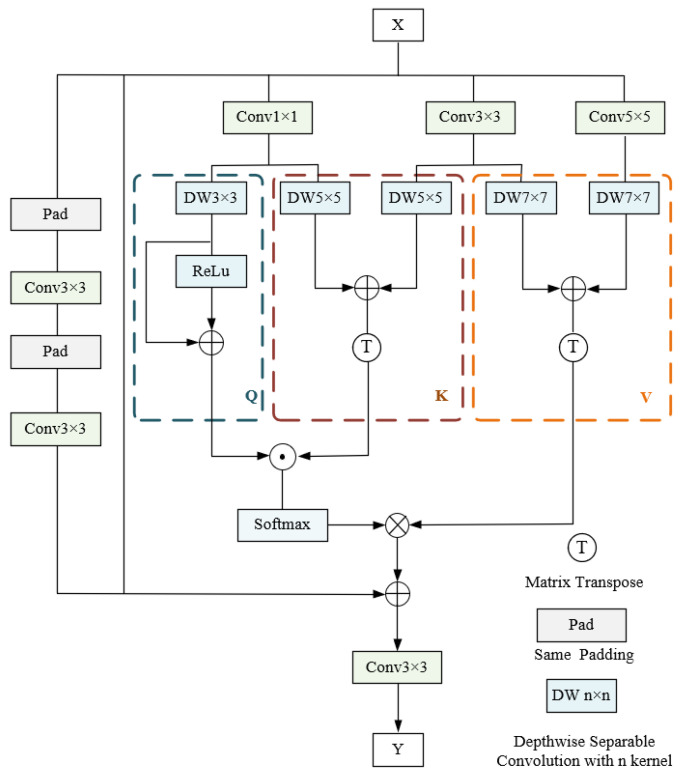
Schematic of DSC-NLA.

Dust in open-pit environments not only occludes target objects but also hinders the extraction of surface texture information [33,34]. As a result, structural features are impaired, owing to the loss of edge information and target–background boundary blurring [35,36]. To address these challenges, an advanced fusion mechanism called DSC-NLA is proposed in this paper, as illustrated in Figure 4. To better capture global spatial dependencies, non-local attention and channel shuffling are combined, which facilitates cross-dimensional feature interaction and calibration.

The corresponding query, key, and value vectors are generated by DSC-NLA, where the concatenated input feature maps are processed by means of depthwise separable convolution. The Q, K, and V in DSC-NLA can be calculated as follows:

(8) $$\begin{array}{cc} \; & Q=\delta \left(f_{dwcon\nu }^{3\times 3}\left(f_{con\nu }^{1\times 1}\left(X\right)\right)\right)\\ \; & K=TP\left(f_{dwcon\nu }^{5\times 5}\left(f_{con\nu }^{1\times 1}\left(X\right)\right)+f_{dwcon\nu }^{5\times 5}\left(f_{con\nu }^{3\times 3}\left(X\right)\right)\right)\\ \; & V=TP\left(f_{dwcon\nu }^{7\times 7}\left(f_{con\nu }^{3\times 3}\left(X\right)\right)+f_{dwcon\nu }^{7\times 7}\left(f_{con\nu }^{5\times 5}\left(X\right)\right)\right) \end{array}$$

where δ denotes the rectified linear unit (ReLU) activation function; TP is the transposition operation; $f_{\mathrm{conv}}^{3\times 3}$, $f_{\mathrm{conv}}^{5\times 5}$, and $f_{\mathrm{conv}}^{7\times 7}$ represent the depthwise convolution operations with kernel sizes of 3 × 3, 5 × 5, and 7 × 7, respectively.

Then, a similarity matrix is established by applying the softmax activation function to the dot product between the query and key vectors. Subsequently, the global spatial dependencies within the feature maps are captured via the multiplication of the matrix by value vectors. As a result, structural features are enhanced through dynamic suppression of dust background noise based on dependencies. The feature map with enhanced structural features (*Y*) can be formulated as follows:

(9) $$Y=f_{con\nu }^{3\times 3}\left(X+Softmax\left(Q\;\cdot \;K\right)\times V+f_{con\nu }^{3\times 3}\left(f_{con\nu }^{3\times 3}\left(X\right)\right)\right)$$

However, the non-local attention mechanism in Formula (9) only focuses on structured information, leading to smaller yet useful features being filtered out during the feature weighting processing. Thus, a feature reuse structure is applied to compensate for the limitation of the non-local attention mechanism. The output of the feature reuse structure is described in Formula (10), integrating the bottleneck of C2f into DSC-NLA for the implementation of feature reuse.

(10) $$F_{1}\left(x_{c}\right)=x_{c}+F_{p}\left(x_{c}\right)$$

where $F_{1}$ denotes the bottleneck after the introduction of DSC-NLA; $x_{c}$ represents the input feature map; and $F_{p}$ is composed of a 1 × 1 convolution, a 3 × 3 convolution, and the integrated DSC-NLA.

Finally, the CSO is introduced to enhance inter-channel information flow, and a bottleneck block with two groups is divided into several subgroups. Then, subgroups are fed to the next layer’s corresponding groups in a shuffled manner to enable efficient cross-group channel interaction.

## 4. Results and Discussion

In this section, lots of comparative and ablation experiments are conducted on the DAWN public dusty dataset and the self-made DOM dataset. All experiments are performed on an Ubuntu 20.04, using the PyTorch 1.10.0 deep learning framework and CUDA 11.3 for computational acceleration, with models trained and validated on an NVIDIA RTX 3080 GPU.

### 4.1. Construction of Dusty Open-Pit Mine Datasets

**Figure 5:**
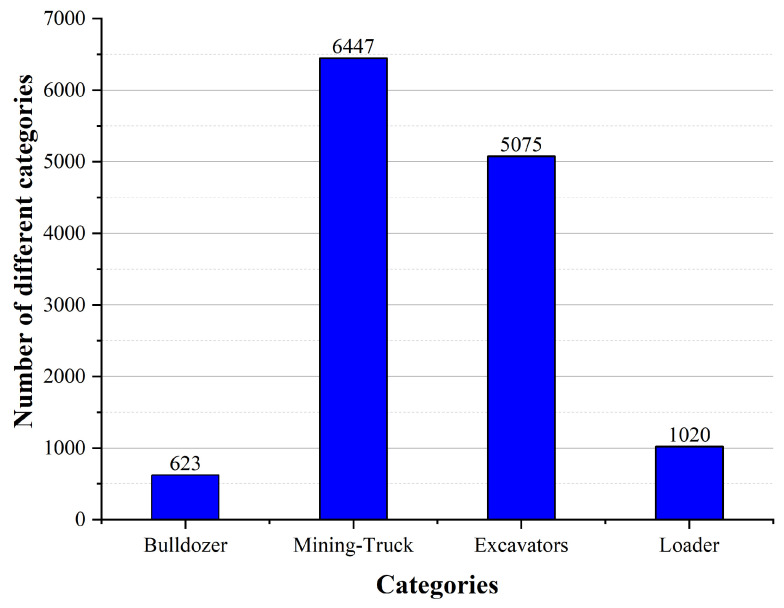
Distribution of different objects in DOM.

The Dusty Open-pit Mine (DOM) data is constructed in this paper for dust-obscured open-pit mining environments. It is composed of field data acquisition, selective sampling from the AutoMine database, and CycleGAN-based data augmentation. To validate a robust object detection algorithm for autonomous mining trucks in dusty conditions, this paper constructs the DOM dataset, which contains 7371 dusty images across four categories and is divided into training and testing sets in an 8:2 ratio. The four categories are Bulldozer, Mining-Truck, Excavators, and Loader. Among these, the sample count for “Bulldozer” is 623, and the numbers of “Mining-Truck”, “Excavators”, and “Loader” instances are 6447, 5075, and 1020, respectively. The scale and distribution of different categories of objects are shown in Figure 5.

The CycleGAN model architecture consists of a ResNet-based generator with six residual blocks and two stride-2 convolutions for downsampling, paired with a 70 × 70 PatchGAN discriminator, implementing three key loss functions: cycle-consistency loss (λ = 10), LSGAN adversarial loss, and identity loss (λ = 0.5). Training was conducted on an unpaired dataset of 7371 real dusty and 7371 synthetic clean images using the Adam optimizer (lr = 0.0002, β1 = 0.5) for 300 epochs with a fixed batch size of 8, maintaining a 1:1 synthetic-to-real data ratio during detector training to preserve CycleGAN output diversity while strictly adhering to the original CycleGAN methodology without architectural modifications or pretraining.

Our framework creates a closed-loop system where CycleGAN generates DOM training data with physically accurate dust patterns. These synthetic data directly shape MBFILNet’s architectural design; specifically, its MBFI-DO and DSC-NLA modules are optimized to handle the characteristic interference patterns present in CycleGAN-generated data. To rule out data leakage, we perform forced bundling of adjacent time-series data, selecting data samples from different time periods as the training set and test set so as to ensure complete independence between the training set and the test set.

### 4.2. Evaluation Metrics

In the object detection experiments on the DOM and the DAWN public dusty cityscape datasets, the models were pretrained for 100 epochs on the COCO dataset. Specifically, the number of epochs and learning rate were set to 200 and 0.01 in the training of the models, and the batch size was set to 16. In addition, mean Average Precision (mAP) is adopted as the evaluation criterion, with a confidence threshold of 0.5. The formulas for precision, recall, AP, and mAP are expressed as follows:

(11) P=TP/(TP+FP)

(12) R=TP/(TP+FN)

(13) $$AP=\int_{0}^{1}P\left(R\right)\;dR$$

(14) $$mAP=\frac{1}{N}\sum_{i\in N}AP_{i}$$

where TP is the number of true-positive bounding boxes with an IoU > 0.5, FP is the number of false-positive bounding boxes with an IoU ≤ 0.5, FN is represents false negatives, and *N* refers to the number of object classes.

### 4.3. Experiments on DOM and DAWN

Comparison experiments with several classic object detection models are conducted in this section, demonstrating the excellent performance of MBFILNet in dusty open-pit mines. Comparison models include the YOLO series, dusty image restoration before detection (DIR), domain adaptation detection (DAD) methods, and enhanced detection algorithms (EDG). All models are trained and tested based on DOM and DAWN, and training is stopped when the model reaches convergence.

**Figure 6:**
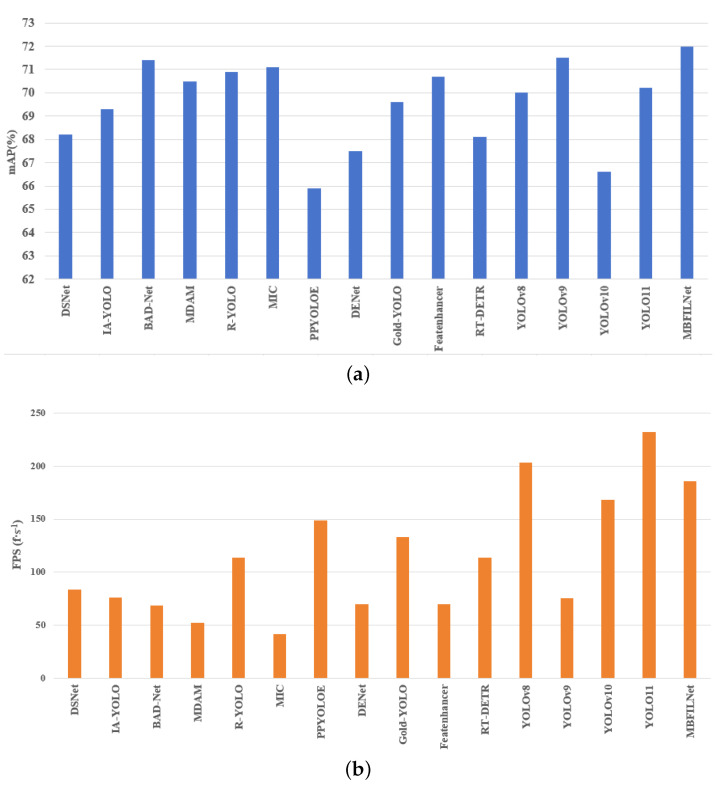
FPS and mAP in different dust conditions. (**a**) mAP; (**b**) FPS.

The mAP and FPS of the object detection models in different dust conditions are shown in Figure 6. As can be seen from Figure 6, compared with mainstream detection methods for adverse weather conditions, MBFILNet achieved the highest detection accuracy. More importantly, while delivering this exceptional precision, MBFILNet also maintains competitive FPS performance, surpassing most existing models. This dual advantage highlights that MBFILNet has achieved an optimal balance between detection accuracy and computational efficiency.

As can be seen from Table 1, compared with DIR, a mAP value of 72.0% is obtained by MBFILNet, which is higher than that of DSNet, IA-YOLO, and BAD-Net by 3.8%, 2.7%, and 0.6%, respectively. Simultaneously, in comparison with the DAD MIC, the mAP of the proposed model is enhanced by 0.9% and 1.1% on the DOM and DAWN datasets, respectively. Furthermore, MBFILNet surpasses the advanced Featenhancer EDG by 1.3% on DOM and 2.6% on DAWN. According to the FPS indicators in Table 1, the FPS values of DIR, DAD, and EDG are substantially lower, rendering them unsuitable for object detection of AMTs. In addition, compared with the base YOLOv8 model, the mAP values of MBFILNet are improved by 2.0% and 3.7% on the DOM and DAWN datasets. MDFILNet achieves best accuracy performance compared to the original models while maintaining efficient operation, with only a modest 17.8 FPS increase in computational cost. The optimal balance between accuracy and processing speed fully meets the real-time requirements of AMTs. Notably, MBFILNet also outperforms the newer YOLO11, which increased mAP by 1.8% on the DOM dataset and 1.7% on the DAWN dataset.

**Figure 7:**
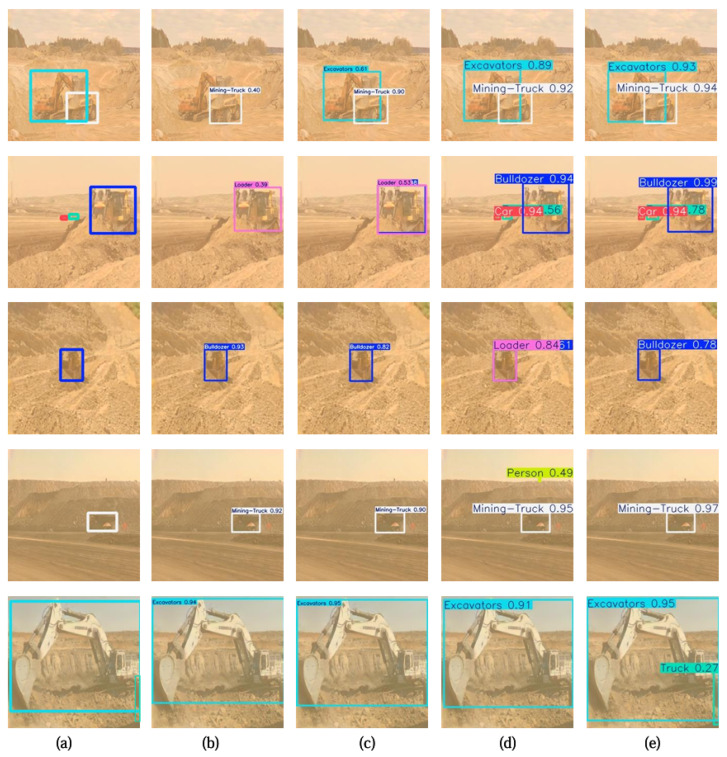
Object detection results with different methods on DOM. (**a**) Ground truth; (**b**) BAD-Net, (**c**) YOLO11; (**d**) YOLOv8; (**e**) MBFILNet.

The detection results of the above models on the DOM datasets are visualized in Figure 7. Although BAD-Net adaptively enhances the input images by eliminating weather-specific information, some relevant target features are inevitably lost during the process. In comparison, YOLO11 effectively enhances multi-scale feature fusion through cross-scale connections and deformable convolutional modules. However, false detections in complex dusty environments occur due to inadequate structural information. Moreover, while multi-scale feature are extracted by the multiple layers of YOLOv8, it cannot overcome severe dust interference. In contrast, MBFILNet achieves better performance by precisely capturing important object details and structural information in dusty environments.

It can be concluded that MBFILNet performs best in various experiments compared with various classic algorithms on the DOM and DAWN datasets. The comparison shows that the introduction of MBFI-DO and DSC-NLA in MBFILNet not only improves detection accuracy but also significantly reduces both false and missed detections, exhibiting stronger overall robustness.

### 4.4. Ablation Study

This section investigates the robustness of each component of the detection method proposed in this paper. All experiments are conducted on the DOM dataset, and the baseline model is built based on YOLOv8, with results shown in Table 2.

**Figure 8:**
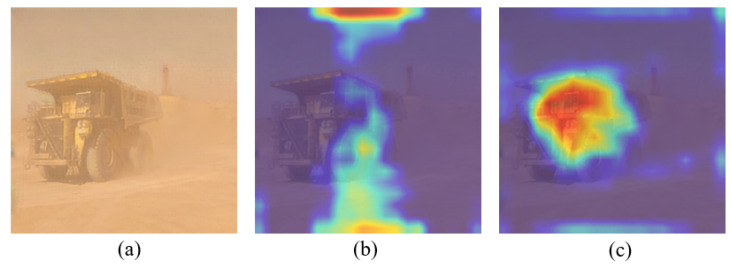
Heatmap visualization comparison with different methods. (**a**) Original image; (**b**) feature Heatmap produced by YOLOv8; (**c**) feature heatmap produced by YOLOv8-MBFI-DO.

As can be seen from Table 2, YOLOv8-MBFI-DO improved mAP by 1.4% compared with YOLOv8. As illustrated in Figure 8, the interference from the dust background is reduced by MBFI-DO under the guidance of semantic information, thereby enhancing the salient feature representation of target objects. This demonstrates that MBFI-DO can improve detection accuracy by focusing on the essential characteristics of objects in open-pit mines affected by dust.

**Figure 9:**
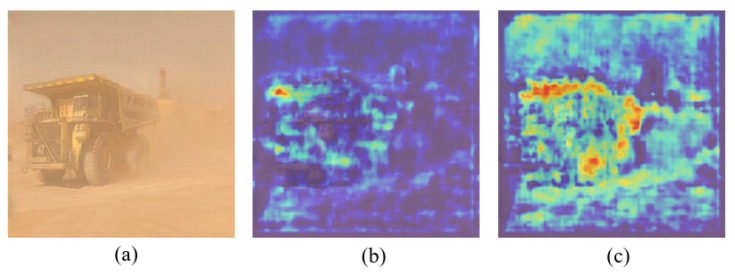
Heatmap visualization comparison with different methods. (**a**) The original image; (**b**) feature heatmap produced using YOLOv8; (**c**) feature heatmap produced by YOLOv8-DSC-NLA.

In addition, mAP values based on YOLOv8-DSC-NLA are improved by 1.6% relative to YOLOv8. As a result, in comparison to standard NLA, DSC-NLA demonstrates better suitability for target detection of AMTs in dusty conditions in comparative ablation studies. Figure 9 verifies that target structural information is enhanced by modeling global spatial long-range dependencies based on DSC-NLA, thereby emphasizing contour boundary features between objects.

Notably, the mAP is increased by 2.0% using the combination of MBFI-DO and DSC-NLA, where local semantic information is enriched to reduce dusty background interference. Furthermore, compared with YOLOv8, the MBFILNet proposed in this paper improves accuracy at the cost of a slightly increased computational load, ensuing the suitability for the mobile deployment of AMTs in dusty environments.

#### 4.4.1. Ablation Experiments on MBFI-DO

In this section, ablation experiments on MBFI-DO are performed to explore the effects of MBFI and DO, the results of which are shown in Table 3.

As can be seen from Table 3, detection performance is enhanced by each component of MBFI-DO. Compared to YOLOv8, MBFI and DO significantly enhance mAP by 1.0% and 0.7% on DOM and 0.7% and 0.8% on DAWN, respectively. In particular, compared with the baseline model with YOLOv8, 1.0% and 2.0% mAP improvements are achieved on the DOM and DAWN datasets due to MBFI. Moreover, the salient feature representations of the targets are better focused under DO guidance, which also reduces the number of parameters.

Through the combined experiments on different pooling operations of GAP and GMP in feature extraction, it can be found that the accuracy improvement is maximized by locating GAP with larger kernel convolution after feature extraction. The reason is that context space information is better focused based on GAP with a larger kernel convolution. Meanwhile, the salient information of the target is more the focus of GMP, which employs gradient-guided feature amplification to suppress non-critical regions. In addition, the GAP and GMP effects of the exchange order achieve the worst performance, with the map decreasing by 2.1% and 3.8% on DOM and DAWN datasets, respectively (Table 4).

#### 4.4.2. Ablation Experiments on DSC-NLA

The experimental results for SE, CA, DAM, and NLA are presented in Table 5. Compared to DAM, NLA significantly enhances mAP by 0.6% and 1.2% on DOM and DAWN, respectively. The increased performance is attributed to the NLA modeling of dependencies between different object directions under the interference of dusty environments.

As shown in the Table 6, compared with STDConv and GhostConv, the depth-separable (DW) convolution applied in this paper not only achieves a reduction in parameters but also improves the accuracy of the mAP on the DOM dataset by 0.8% and 0.4%, respectively, which is attributable to the fact that DW convolution achieves spatial filtering of each input channel separately, avoiding the weight coupling between channels observed in standard convolution.

### 4.5. Robustness Evaluation

In the DOM dataset, we categorized the test sets into three levels (clear, light dust, and heavy dust) for dust conditions in open-pit mining environments. Clear conditions with visibility exceeding 80 m represent normal operations. Light dust conditions occur when visibility ranges between 30 and 80 m, indicating moderately challenging working environments. Heavy dust conditions emerge when visibility drops below 30 m. Based on the above standards, the test proportions of clear weather, light dust, and heavy dust are 37%, 52%, and 11%, respectively.

**Figure 10:**
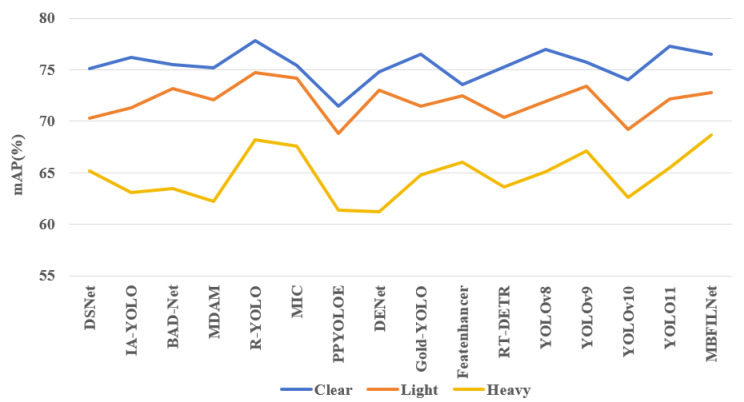
mAP under different dust conditions.

The mAP curves in different dust scenarios are shown in Figure 10. As can be found from Figure 10, MBFILNet demonstrates the most outstanding detection performance under heavy dust conditions, fully demonstrating the robustness of MBFILNet against interference in dusty environments. It is worth noting that as the dust becomes more severe, the detection performance of existing algorithms shows a significant downward trend. This phenomenon further verifies the fact that dust conditions could exacerbate the difficulty of AMT target detection.

Additionally, the experimental results demonstrate MBFILNet’s superior robustness under challenging dust conditions. As indicated in Table 7, MBFILNet achieves the highest detection accuracy of 68.7% mAP in heavy dust environments, outperforming all comparable real-time methods. Notably, it maintains a significant 0.5% mAP improvement over R-YOLO while delivering comparable processing speeds of 185.6 FPS versus 113.7 FPS. While conventional YOLO-series detectors exhibit excellent computational efficiency, they show notable performance degradation in heavy dust conditions, with mAP values of YOLOv9 approximately 1.6% lower than those of MBFILNet. Compared with YOLO-series models, the proposed MBFILNet model successfully addresses the common trade-off between speed and accuracy in dust-obscured environments, achieving both real-time processing capabilities and superior detection robustness.

Table 8 presents the detailed performance metrics of MBFILNet over five independent training runs. The results show minimal fluctuation across runs, with run 3 achieving the highest mAP value of 72.2% on DOM and run 4 showing the best performance of 56.1% on DAWN. The calculated average mAP values of 72.02% and 55.88% confirm the stability of our approach, while the narrow standard deviations of ±0.2% and ±0.3% further substantiate the reproducibility of these improvements. These comprehensive measurements address potential concerns about performance variance and validate the reliability of our reported results.

Furthermore, in order to verify the generalization ability of MBFILNet, we also conducted experimental comparisons on the public non-dust KITTI dataset. A comparison of the performance of object detection methods on the KITTI dataset is shown in Table 9. In Table 9. With a precision of 94.2% and a recall of 88.6%, MBFILNet achieves the highest mAP value of 93.4% among all evaluated methods, surpassing Faster R-CNN at 5.2%, YOLOv8 at 0.6%, and YOLOv9 at 1.0%. In terms of processing speed, MBFILNet maintains excellent efficiency at 185.6 FPS, significantly outperforming Transformer-based approaches like RT-DETRv2-R18, achieving an mAP value of 90.4% at only 28.9 FPS. This comprehensive evaluation demonstrates MBFILNet as a particularly effective solution for autonomous driving applications where both detection accuracy and real-time performance are critical requirements. The comparative analysis of object detection methods on the KITTI dataset demonstrates MBFILNet’s superior generalization across different scenarios.

As shown in Table 9, the comparative analysis of object detection methods on the KITTI dataset demonstrates MBFILNet’s superior performance across multiple metrics. With a precision of 94.2% and recall of 88.6%, MBFILNet achieves the highest mAP value of 93.4% among all evaluated methods, surpassing Faster R-CNN at 5.2%, YOLOv8 at 0.6%, and YOLOv9 at 1.0%. In terms of processing speed, MBFILNet maintains excellent efficiency at 185.6 FPS, significantly outperforming Transformer-based approaches like RT-DETRv2-R18, which achieves an mAP value of 90.4% at only 28.9 FPS. This comprehensive evaluation positions MBFILNet as a particularly effective solution for autonomous driving applications where both detection accuracy and real-time performance are critical requirements. The above comparative experiments also demonstrate the outstanding performance of MBFILNet on a non-dusty dataset, highlighting the generalization ability of MBFILNet.

## 5. Conclusions

To improve detection accuracy in dusty environments, an efficient object detector for AMTs in dusty environments called MBFILNet is proposed in this paper, which incorporates MBFI-DO and DSC-NLA modules. MBFI-DO enhances discriminative features in target regions and integrated semantic information across multiple levels. DSC-NLA captures global spatial long-range dependencies based on pixel correlations. Meanwhile, a feature fusion strategy is implemented to aggregate diverse receptive-field representations, enhancing multi-scale object detection capability.

According to abundant experiments and comparison with the latest methods on self-made and public datasets, complemented by extensive validation and data analysis, the proposed MBFILNet performs better in AMT object detection under dusty conditions. Notably, MBFILNet achieved an mAP of 72.0% on our self-made DOM dataset and 55.8% on the public DAWN dataset, representing significant improvements of 2.0% and 3.7%, respectively, over the baseline YOLOv8 model. The superior capability of MBFILNet is demonstrated by these gains, addressing the challenges of low object detection accuracy caused by hard extraction of salient represent feature and edge information in dusty backgrounds.

Although the proposed MBFILNet performs robust detection in dusty environments, it performance in extremely dust-laden environments is unsatisfied. Extremely dust-laden environments include environments with extremely high dust concentrations and visibility lower than 5 m, as well as sandstorm conditions. Furthermore, the unique working environment of open-pit mines is not only associated with significant dust problems but also frequent complex adverse conditions such as low light, rain, and snow. When these environmental factors occur simultaneously with dust, more extreme multimodal interference scenarios are formed, posing even greater challenges to the perception systems of unmanned mining trucks. As extremely dust-laden conditions severely degrade image quality and cause significant information loss, target discriminability should be enhanced by means of multi-sensor fusion in the future.

## Figures and Tables

**Table 1:** Quantitative comparison of results with those of mainstream detection methods on the DOM and DAWN datasets. Bold values indicate the best performance for each metric.

Method		FPS (f s^−1^)	Datasets					
			DOM					DAWN
			Bulldozer	Mining-Truck	Excavators	Loader	mAP (%)	mAP (%)
DIR	DSNet [14]	83.4	60.8	75.3	70.1	66.6	68.2	51.8
	IA-YOLO [15]	76.2	61.9	76.7	71.2	67.4	69.3	53.1
	BAD-Net [37]	68.5	63.7	78.9	73.3	**69.7**	71.4	54.6
DAD	MDAM [38]	52.3	62.9	77.8	72.3	69.0	70.5	52.3
	R-YOLO [17]	113.7	63.2	78.3	72.7	69.4	70.9	55.1
	MIC [39]	41.6	63.5	78.6	72.9	69.6	71.1	54.7
EDG	PPYOLOE [40]	149.1	58.7	72.6	67.8	64.5	65.9	48.1
	DENet [41]	69.9	60.2	74.5	69.3	66.0	67.5	49.8
	Gold-YOLO [42]	132.9	62.1	76.8	71.5	68.0	69.6	51.7
	Featenhancer [43]	69.5	63.1	77.9	72.6	69.2	70.7	53.2
	RT-DETR [44]	114.0	60.7	75.2	70.0	66.5	68.1	50.2
YOLO Series	YOLOv8 [45]	232.1	62.4	77.1	71.9	68.6	70.0	52.1
	YOLOv9 [46]	75.5	63.8	78.7	**73.4**	69.9	71.5	54.0
	YOLO10 [47]	168.4	59.4	73.4	68.5	65.1	66.6	53.9
	YOLO11 [48]	**271.5**	62.6	77.4	72.1	68.7	70.2	54.1
Ours	MBFILNet	185.6	**64.7**	**79.6**	73.2	69.5	**72.0**	**55.8**

**Table 2:** Ablation experiments on different module combinations. Bold values indicate the best performance for each metric.

Method	Module		Metrics		
	MBFI-DO	DSC-NLA	Para (M)	FLOPs (G)	DOM mAP (%)
YOLOv8			**3.2**	**8.7**	70.0
	√		3.6	9.5	71.4
		√	3.3	9.1	71.6
Ours	√	√	3.9	9.8	**72.0**

**Table 3:** Comparison and verification of MBFI-DO performance. Bold values indicate the best performance for each metric.

Module			Metrics			
MBFI	DO	ADD	Para (M)	FLOPs (G)	DOM mAP (%)	DAWN mAP (%)
√			3.4	9.2	71.0	54.1
	√		**3.3**	**8.8**	70.7	52.9
		√	3.7	10.2	67.7	50.8
√		√	4.2	11.7	68.1	51.1
√	√		3.6	9.5	**71.4**	**55.0**

**Table 4:** Comparison and verification of MBFI performance. Bold values indicate the best performance for each metric.

Module				Metrics	
Branch1		Branch2		DOM	DAWN
GAP	GMP	GAP	GMP	mAP (%)	mAP (%)
√		√		70.8	53.9
	√		√	70.6	51.7
	√	√		68.9	50.3
√			√	**71.0**	**54.1**

**Table 5:** Comparison and verification of DSC-NLA performance. Bold values indicate the best performance for each metric.

Module				Metrics	
SE	CA	DAM	NLA	DOM mAP (%)	DAWN mAP (%)
√				68.3	50.2
	√			69.8	51.4
		√		70.2	52.1
			√	**70.8**	**53.3**

**Table 6:** Comparison and verification of DWConv performance. Bold values indicate the best performance for each metric.

Module			Metrics			
STDConv	Ghost	DW	Para (M)	FLOPs (G)	DOM mAP (%)	DAWNmAP (%)
√			4.9	10.7	70.8	53.3
	√		4.2	10.1	71.2	53.8
		√	3.6	9.5	**71.6**	**54.1**

**Table 7:** Quantified object detection performance under graded dust condition on DOM with mainstream detection methods. Bold font indicates the optimal value.

Category	Method	FPS (f s^−1^)	DOM mAP(%)		
			Clear	Light	Heavy
DIR	DSNet [14]	83.4	75.1	70.3	63.2
	IA-YOLO [15]	76.2	76.2	71.3	63.1
	BAD-Net [37]	68.5	**78.5**	73.2	66.5
DAD	MDAM [38]	52.3	72.2	72.1	65.2
	R-YOLO [17]	113.7	77.8	72.7	66.2
	MIC [39]	41.6	78.2	73.0	66.6
EDG	PYYOLOE [40]	149.1	73.5	68.8	61.4
	DENet [41]	69.9	74.8	70.0	63.2
	Gold-YOLO [42]	132.9	76.5	71.5	64.8
	Federalancer [43]	69.5	77.6	72.5	66.0
	RT-DETR [44]	114.0	75.3	70.4	63.6
YOLO Series	YOLOv8 [45]	232.1	77.0	71.9	65.1
	YOLOv9 [46]	75.5	70.7	**73.4**	67.1
	YOLOv10 [47]	168.4	74.0	69.2	62.6
	YOLO11 [48]	**271.5**	77.3	72.2	65.5
Ours	MBFTLNet	185.6	76.5	72.8	**66.7**

**Table 8:** Robustness evaluation across multiple runs.

Runs	Metrics	
	DOM mAP (%)	DAWN mAP (%)
1	71.8	55.7
2	72.0	55.9
3	72.2	55.8
4	71.8	56.1
5	72.3	55.9
Average	72.02	55.88

**Table 9:** Comparative Performance Evaluation of Object Detection Methods on the KITTI dataset. Bold font indicates the optimal value.

Module	Precision (%)	Recall (%)	mAP (%)	FPS
SSD	89.7	64.4	72.4	59
Faster R-CNN	86.5	74.3	88.2	70.5
RT-DETR-R18	84.7	80.5	88.2	39.4
RT-DETRv2-R18	87	85.1	90.4	28.9
LKStar-YOLO	84.6	78.1	85.4	164.5
YOLOV8	92.9	88.1	92.8	**217.4**
YOLOV9	93.1	88.4	92.4	70.7
YOLOV10	93	85.1	92.2	157.6
YOLO11	92.6	87.3	92.4	90.9
CR-YOLO	**94.7**	86.1	92.2	78.4
BML-YOLO	92.6	85.1	93.0	168.7
MBFILNet	94.2	**88.6**	**93.4**	185.6

## Data Availability

The data presented in this study are available from the corresponding author upon request.
